# Novel Zwitterionic Hydrogels with High and Tunable Toughness for Anti-Fouling Application

**DOI:** 10.3390/gels11080587

**Published:** 2025-07-30

**Authors:** Kefan Wu, Xiaoyu Guo, Jingyao Feng, Xiaoxue Yang, Feiyang Li, Xiaolin Wang, Hui Guo

**Affiliations:** 1 State Key Laboratory of Quality Research in Chinese Medicine, Faculty of Chinese Medicine, Macau University of Science and Technology, Macao 999078, China; 13655287889@163.com (K.W.); 13775010950@163.com (J.F.);; 2 School of Chemical Engineering and Technology, Sun Yat-sen University, Zhuhai 519082, China

**Keywords:** zwitterionic hydrogels, high strength, mechanical properties, thermo-responsiveness, anti-fouling

## Abstract

Zwitterionic hydrogels have emerged as eco-friendly anti-fouling materials owing to their superior hydration-mediated resistance to biofouling. Nevertheless, their practical utility remains constrained by intrinsically poor mechanical robustness. Herein, this study proposes a novel strategy to develop novel tough zwitterionic hydrogels by freezing the gels’ polymer network. As a proof of concept, a zwitterionic hydrogel was synthesized via copolymerization of hydrophobic monomer phenyl methacrylate (PMA) and hydrophilic cationic monomer *N*-(3-dimethylaminopropyl) methacrylamide (DMAPMA), followed by post-oxidation to yield a zwitterionic structure. At service temperature, the rigid and hydrophobic PMA segments remain frozen, while the hydrophilic zwitterionic units maintain substantial water content by osmotic pressure. Synergistically, the zwitterionic hydrogel achieves robust toughness and adhesiveness, with high rigidity (66 MPa), strength (4.78 MPa), and toughness (2.53 MJ/m^3^). Moreover, the hydrogel exhibits a distinct temperature-dependent behavior by manifesting softer and more stretchable behavior after heating, since the thawing of the gel network at high temperatures increases segmental mobility. Therefore, it achieved satisfactory adhesiveness to substrates (80 kPa). Additionally, the hydrogel demonstrated remarkable anti-fouling performance, effectively suppressing biofilm formation and larval attachment. In summary, this work opens up promising prospects for the development of zwitterionic hydrogels with high application potential.

## 1. Introduction

The process by which micro- and macro-organisms colonize artificial marine substrates is known as marine biofouling [1,2,3]. Biofouling poses severe economic and operational challenges by increasing hydrodynamic drag and fuel consumption in maritime transport, accelerating material corrosion through microbial activity, and impairing the efficiency of heat exchangers and water treatment systems [4,5,6,7]. Additionally, biofouling threatens marine ecosystems by facilitating invasive species spread and compromises medical devices by promoting persistent infections and antibiotic resistance [8,9,10,11]. To address this concern, recent years have witnessed rapid advancements in anti-fouling coatings, among which hydrogel-based materials with high water content have emerged as a research hotspot due to their ability to form favorable interfacial layers with fouling organisms during service [12,13]. Compared to other anti-fouling coatings, hydrogels demonstrate distinct advantages, including low surface energy, non-toxicity, environmental protection, and high levels of smoothness and softness [14,15,16]. In hydrogel-based anti-fouling materials, high ionic content typically serves as the critical determinant for their fouling-resistant performance [17,18]. Among these, zwitterionic hydrogels stand out due to their unique molecular structure containing equal cationic and anionic groups [19,20,21]. Abundant in ionic units, zwitterionic hydrogels can form a stable hydration interface, which acts as a protective film and can prevent the adsorption and deposition of biomolecules such as proteins and microorganisms [22]. Additionally, zwitterionic hydrogel coatings exert a positive effect by reducing the attachment of marine organisms, thereby reducing the maintenance cost and energy consumption of ships [23]. Therefore, zwitterionic hydrogel coatings can effectively reduce this biological contamination, reduce the risk of infection, and prevent the occurrence of bio-fouling.

Despite rapid advancements, the limitations of zwitterionic hydrogels are becoming increasingly apparent. While these materials demonstrate exceptional anti-fouling performance through their high ionic density and superior hydration capacity, they often suffer from inherent weaknesses in mechanical performance, making them unable to withstand large mechanical forces or even prone to deformation and damage. For instance, conventional zwitterionic hydrogels typically exhibit tensile strengths ranging from merely tens of kPa, which falls significantly short of the mechanical requirements for most practical applications [24,25]. Consequently, recent years have witnessed a surge in research on innovative strategies for toughening zwitterionic hydrogels. A common strategy to enhance mechanical strength is to ameliorate the topological structure. For example, Zhang et al. successfully prepared double-network zwitterionic hydrogels through novel hybrid ionic–covalent crosslinked zwitterionic hydrogels [26]. This innovation in topological structure endows the hydrogel with an efficient energy dissipation mechanism, thereby achieving high tensile strength (0.69 MPa) and a strong elastic modulus (0.28 MPa). Nevertheless, current zwitterionic hydrogels still fail to meet the stringent requirements for long-term service conditions, which typically require a tensile stress over MPa [27].

The intrinsically weak mechanical properties of zwitterionic hydrogels directly result from their unique network structure [28,29,30]. The high ionic density induces excessive water absorption and enhanced chain mobility, maintaining the polymer matrix in a highly swollen state [31,32]. This structural characteristic, while beneficial for anti-fouling performance, inevitably compromises mechanical integrity [33]. Recent studies suggest that controlled immobilization of polymer networks through physical or chemical freezing strategies could effectively enhance the mechanical strength of gels without sacrificing functionality [34,35,36,37]. Furthermore, this characteristic imparts the material with temperature-responsive behavior. However, such network freezing approaches have not yet been successfully applied to zwitterionic hydrogel systems, creating a significant research gap that needs to be addressed to enable their practical applications.

**Figure 1:**
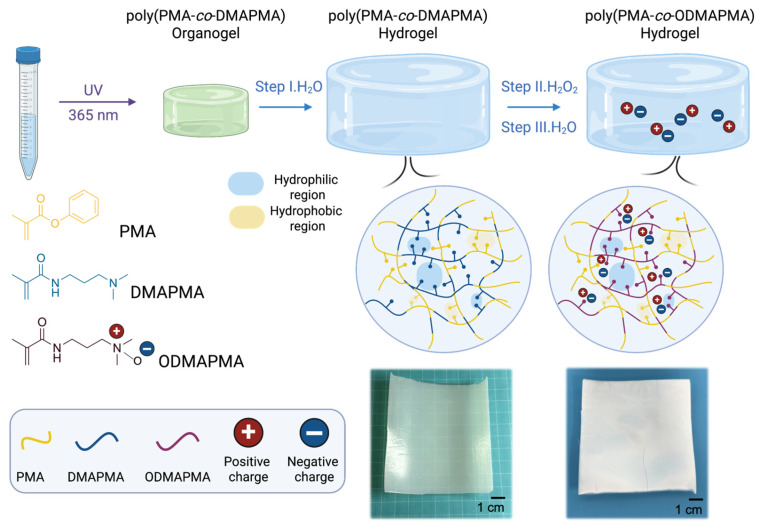
Schematic illustration of fabrication process of poly(PMA-*co*-ODMAPMA) hydrogel. Images on lower right show poly(PMA-*co*-DMAPMA) hydrogel after step I and step II.

Hereby, this study reports a novel tough zwitterionic hydrogel created by freezing the polymer network through molecular structure design. To clarify this concept specifically, the poly(PMA-*co*-ODMAPMA) hydrogel was first prepared with a hydrophobic and rigid structure provided by phenyl methacrylate (PMA) and a hydrophilic monomer, dimethylaminopropyl methacrylamide (DMAPMA). Afterward, the cationic amine group in DMAPMA was oxidized to yield a zwitterionic structure (Figure 1). More specifically, the PMA phase separates to afford a rigid structure and gives rise to robust mechanical properties, with high rigidity (66 MPa), strength (4.78 MPa), and toughness (2.53 MJ/m^3^). Moreover, the zwitterionic hydrogel exhibits a distinct temperature-dependent behavior, manifesting significant temperature-responsive mechanical performance and high adhesiveness with the thawing of the network. In addition, the zwitterionic hydrogels demonstrate significant anti-fouling capabilities, effectively inhibiting biofilm formation and preventing larval attachment. The combination of temperature-responsive behavior and robust anti-fouling performance highlights the versatility of these hydrogels for multifunctional applications.

## 2. Results and Discussion

### 2.1. Preparation and Characterization of Poly(PMA-co-ODMAPMA) Hydrogel

The poly(PMA-*co*-ODMAPMA) hydrogel was prepared through a straightforward procedure. Initially, a mixture of PMA and DMAPMA as well as a small amount of crosslinkers were subjected to 8 h ultraviolet irradiation to form the crosslinked network structure. Subsequently, the gel underwent solvent exchange in pure water to remove unreacted monomers and organic impurities while simultaneously inducing phase separation. In particular, the hydrophobic PMA units underwent phase separation, while the highly hydrophilic DMAPMA chains absorbed substantial amounts of water through osmotic pressure, thereby maintaining the hydrogel’s hydration capacity. Then, the hydrogels were subjected to oxidation by H_2_O_2_, which turned the DMAPMA into a zwitterionic structure (ODMAPMA). Then, the material was reswelled in pure water until the swelling equilibrium was reached to yield a poly(PMA-*co*-ODMAPMA) hydrogel.

**Figure 2:**
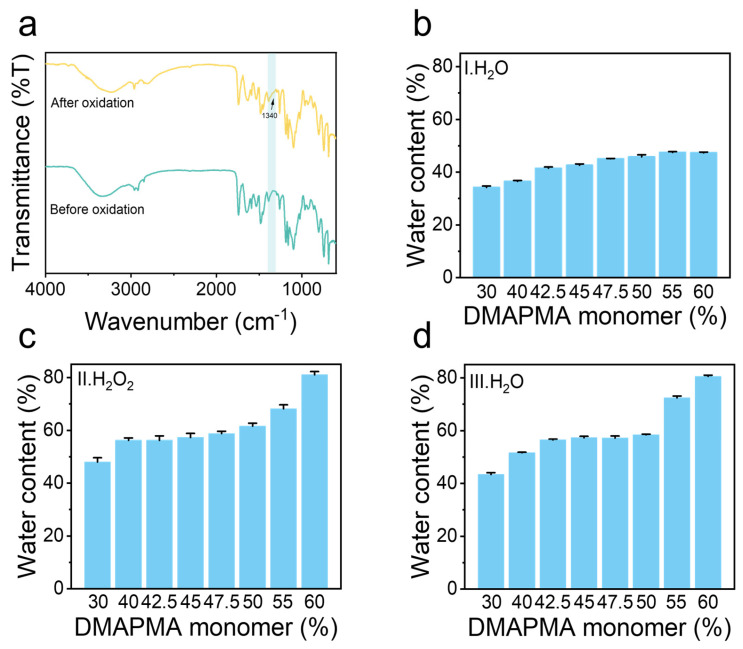
Characterization of poly(PMA-*co*-ODMAPMA) hydrogel. (**a**) Fourier transform infrared spectra of hydrogels at 4000–500 cm^−1^. (**b**) Water content of poly(PMA-*co*-DMAPMA) hydrogel after step I with different DMAPMA monomer ratios, (**c**) poly(PMA-*co*-ODMAPMA) after step II with different DMAPMA monomer ratios, and (**d**) water content of poly(PMA-*co*-ODMAPMA) hydrogel after step III with different DMAPMA monomer ratios. Three steps (I, II, III) are illustrated in Figure 1. Data are expressed as means ± SD, n = 3.

First, the FITR(Nicoletis 50-Raptir spectrometer, Thermo Scientific, Waltham, MA, USA) was used to investigate the chemical structure of the poly(PMA-*co*-ODMAPMA) hydrogel. Figure 2a shows the characteristic peaks of poly(PMA-*co*-ODMAPMA) hydrogel. Specifically, the spectrum absorption peaks of the (C-N) at 1020–1100 cm^−1^ and (N-CH_3_) at 1450 cm^−1^ indicate the copolymerization of the materials. Additionally, when comparing the infrared spectra before and after oxidation, the shift in the C-N stretching vibration was attributed to the oxidation of the tertiary amine group to an N-oxide, indicating successful oxidation. Meanwhile, elemental analysis was conducted to further characterize the oxidation degree of the samples. Judging from the elemental ratios of O/C and O/H, a high conversion rate (92.44%) was obtained from the oxidation reaction.

In addition, the equilibrated water content of the hydrogel also confirmed the successful synthesis process. As shown in Figure 2b, the hydrogels demonstrated a low water content (30–50%) even after swelling until equilibrium. This phenomenon arises from an excess of hydrophobic groups (PMA) in the material coupled with the weak osmotic pressure exerted by the hydrophilic groups (DMAPMA). Moreover, the equilibrated water content demonstrated an evident positive correlation with the DMAPMA ratio. Interestingly, the water content of the hydrogel experienced a remarkable enhancement after oxidation among all groups. This is reasonable since more ionic groups were generated after oxidation, which led to an increase in osmotic pressure that brought more water inside the hydrogel (Figure 2c). Therefore, this further proved the successful oxidation process. After that, the swelling ratio of the hydrogel remained almost constant after being re-immersed in water (Figure 2d).

### 2.2. Mechanical Properties of Poly(PMA-co-ODMAPMA) Hydrogel

**Figure 3:**
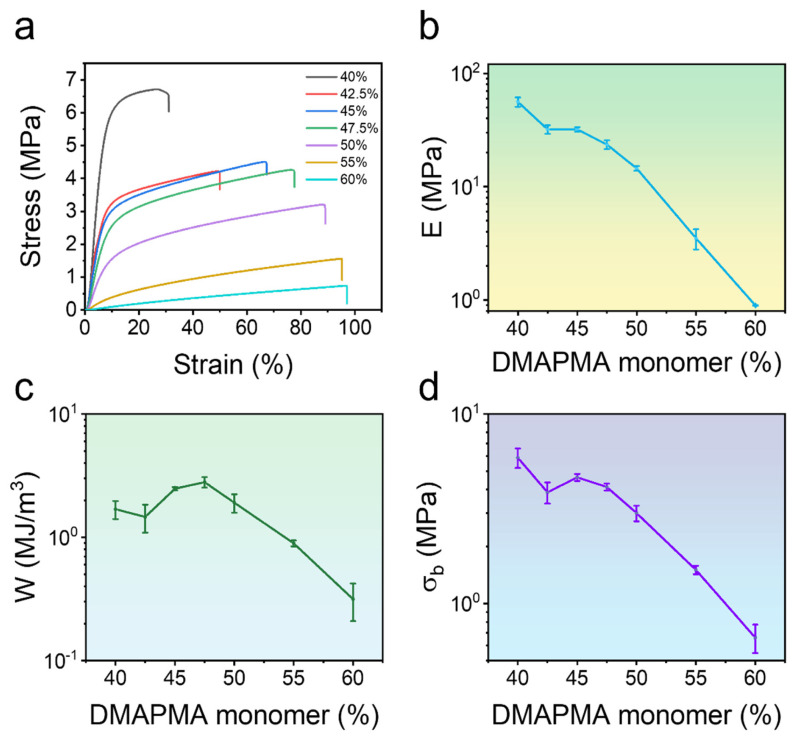
Mechanical properties of poly(PMA-*co*-ODMAPMA) hydrogel at room temperature. (**a**) Uniaxial tensile stress–strain curves with different DMAPMA monomer ratios. (**b**) Young’s modulus, (**c**) fracture energy, and (**d**) fracture stress of different DMAPMA monomer ratios. Data are expressed as means ± SD, n = 3.

Building upon the successful fabrication of the hydrogel, this study conducted quantitative characterization of its critical mechanical properties, including elastic modulus and fracture stress, utilizing tensile tests. Since both properties are essential in the preparation of hydrogels, a pronounced correlation between composition and mechanical performance is revealed in Figure 3. When the hydrophilic monomer content was 40%, the zwitterionic hydrogel exhibited a rigid and brittle mechanical behavior, demonstrating limited fracture tensile elongation. The material displayed a Young’s modulus (*E*) of 66 ± 9 MPa, fracture stress (σ_b_) of 5.96 ± 0.70 MPa, and relatively low tensile work (W) of merely 1.69 ± 0.27 MJ/m^3^. As the proportion of hydrophilic monomer DMAPMA increased, the hydrogel exhibited enhanced elongation and toughness, albeit with a slight reduction in strength. In short, the experiment revealed an inverse correlation between the DMAPMA ratio and tensile strength, with values decreasing from 6.5 MPa to 0.6 MPa as the additive concentration increased, and the tensile strain increasing from 30% to 98% at a constant crosslinking density. Notably, the tensile strength reached a maximum value of 2.48 MJ/m^3^ at a DMAPMA content of 45%. Therefore, it was chosen as a model system for further investigation.

The dependence of important monomers on the mechanical behavior of hydrogels deserves further investigation to disclose the mechanisms behind it. According to previous studies with similar structures [38,39,40], phase separation occurs inside the hydrogel, forming hydrophobic and hydrophilic regions. Indeed, the white appearance of the hydrogel in Figure 1 also supports this point. Specifically, the majority of PMA forms a rigid hydrophobic micro-region, which maintains the hydrogels in a frozen state and serves as a load-bearing phase. Meanwhile, a tiny amount of ODMAPMA, as well as the absorbed water in the PMA regions, serves as a plastificizer; all of this combines to achieve a balance between strength and rigidity. Hence, the energy is effectively dissipated through the freezing of the polymer network. Moreover, another hydrophilic phase, constructed mainly by ODMAPMA and water, contributes to the high hydration level with osmotic pressure.

**Figure 4:**
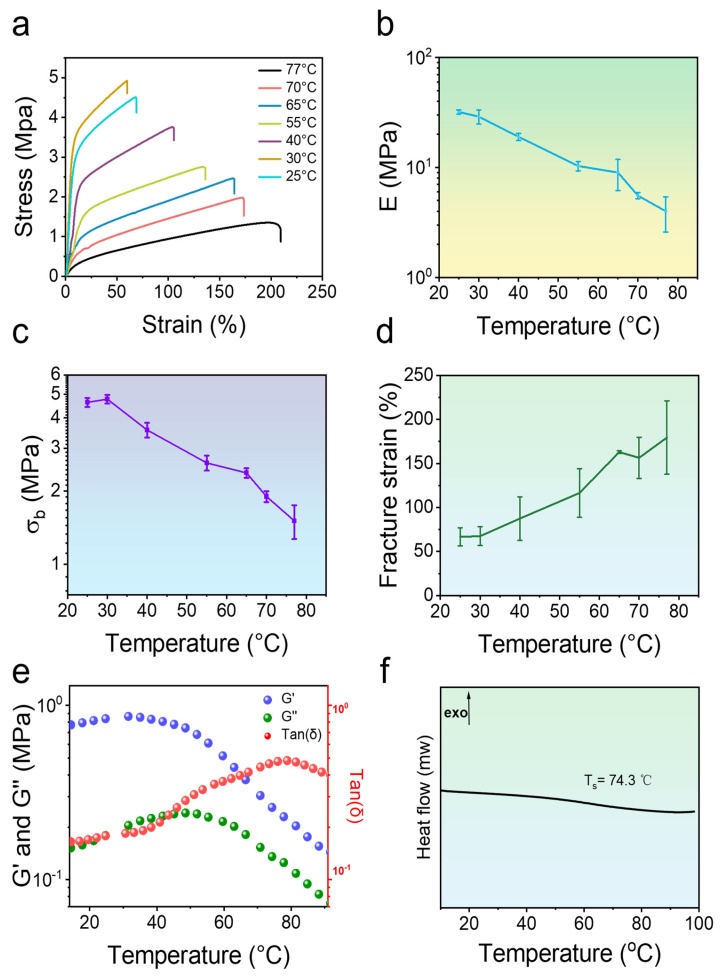
Thermo-responsive mechanical properties of poly(PMA-*co*-ODMAPMA) hydrogel. (**a**) Uniaxial stretching curves of hydrogels at different temperatures. (**b**) Young’s modulus, (**c**) fracture stress (σ_b_), and (**d**) fracture strain of poly(PMA-*co*-ODMAPMA) hydrogel at different temperatures. (**e**) Temperature scan analysis of loss factor (Tanδ), elastic shear storage (G′), and loss shear modulus (G″) of hydrogel. Poly(PMA-*co*-ODMAPMA) hydrogels are prepared with 45% DMAPMA feed ratio. (**f**) DSC curves of Poly(PMA-*co*-ODMAPMA) hydrogels upon heating (heating rate = 10 °C·min^−1^). Exo represents exothermic. Data are expressed as means ± SD, n = 3.

To validate the hypothesis of the frozen network, uniaxial tensile tests were carried out by varying the experimental temperatures. Since the motion of chain segments intensifies with increasing temperatures, this should lead to significant softening effects with a lower rigidity. Figure 4a–d demonstrate the thermo-responsive softening behavior of the hydrogel under large-scale tensile performance. Evidently, the results revealed pronounced temperature-dependent characteristics. Upon heating from an ambient temperature (25 °C) to 77 °C, significant softening and enhanced extensibility of the hydrogel appeared. The Young’s modulus of the hydrogel decreased dramatically, from about 32 ± 0.4 MPa to about 4.0 ± 1.0 MPa. Similarly, the fracture stress of the hydrogel decreased from 4.78 ± 0.14 MPa to 1.50 ± 0.24 MPa, showing a significant temperature dependence. In contrast, the fracture strain showed an increasing trend with increasing temperature, from 67.5 ± 10% at 30 °C to 179.5 ± 40% at 77 °C. This phenomenon can be attributed to the dissociation of the polymer network and the consequent increase in chain segment mobility.

This temperature-sensitive property was further verified in small-scale viscoelastic experiments by rheological tests (Figure 4e). The loss factor Tanδ, an important characteristic of viscoelasticity, exhibits a broad peak at about 78 °C, which corresponds to the softening temperature of the hydrogel. Importantly, this temperature is higher than room temperature, thus ensuring high rigidity under service conditions. Hence, it is reasonable to show the plastic behavior of the gel at room temperature. Moreover, the hydrogel has a high modulus below 50 °C. Upon heating, the storage modulus G′ and the loss modulus G″ decreased significantly at the same time. To further verify the frozen structure, the thermodynamic properties of the hydrogel were also determined through Differential Scanning Calorimetry (DSC) experiments. As shown in Figure 4f, the softening temperature of the gel is 74.3 °C, which is close to that obtained by the rheological test upon measuring the viscoelastic performance. All these results effectively proved that the network of the hydrogel is thawed upon heating above this specific temperature, since the chain segments are thawed.

### 2.3. Adhesion Properties of Poly(PMA-co-ODMAPMA) Hydrogel

To fulfill the requirements of practical applications, adhesive properties constitute another critical performance parameter [41]. Nevertheless, achieving facile and universal adhesion between zwitterionic hydrogels and diverse substrates remains a significant challenge. Indeed, a fundamental conflict exists between the anti-fouling performance, enabled by the non-adhesive nature of zwitterionic hydrogels, and the necessity for strong interfacial adhesion to substrates. Poor adhesion primarily stems from the extensive hydration of the functional groups involved in the bonding process, which substantially reduces the availability of effective binding sites. Consequently, the interfacial interactions between zwitterionic hydrogels and target surfaces are markedly diminished, leading to a pronounced deterioration in adhesive performance [42]. Since our hydrogel exhibits significant thermal softening behavior, we can draw inspiration from the construction methods of hot-melt adhesives in engineering by achieving strong adhesion by first heating and then cooling. At elevated temperatures, the hydrogel’s low modulus allows it to achieve excellent contact with the substrate, enabling effective adhesion through physical interactions and interlocking. Upon cooling, the material’s mechanical properties are significantly enhanced, further improving its adhesive performance.

**Figure 5:**
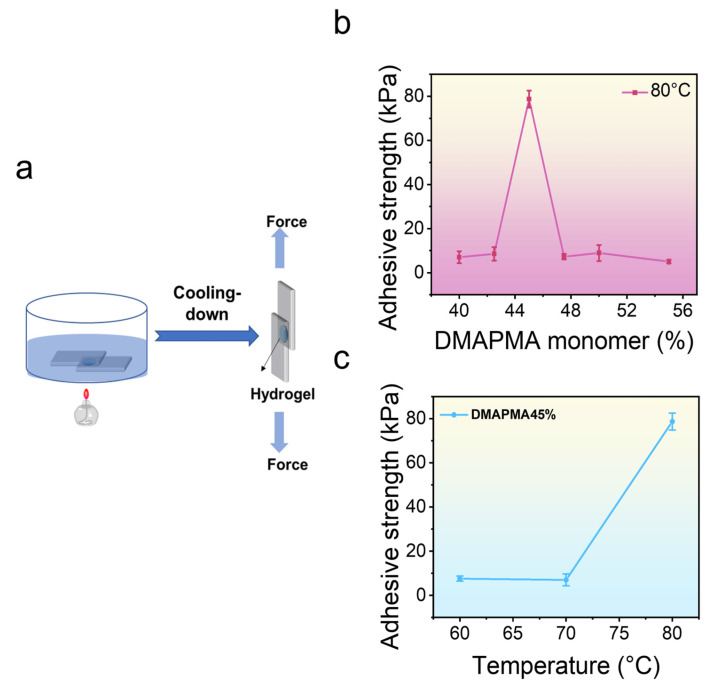
Adhesion properties of poly(PMA-*co*-ODMAPMA) hydrogel. (**a**) Schematic illustration of adhesion properties of poly(PMA-*co*-ODMAPMA) hydrogel. (**b**) Adhesion properties of poly(PMA-*co*-ODMAPMA) hydrogel with different DMAPMA monomer ratios. (**c**) Adhesion properties of poly(PMA-*co*-ODMAPMA) hydrogel at different temperatures. Glass substrates were chosen as model system. Data are expressed as means ± SD, n = 3.

To evaluate the adhesive performance of the hydrogel, shear testing was conducted to experimentally investigate its adhesion to glass substrates (Figure 5a). As shown in Figure 5b, the adhesion of a series of poly(PMA-*co*-ODMAPMA) hydrogels varied significantly. Among them, hydrogels with an intermediate monomer ratio demonstrated optimal adhesive performance. With an initial DMAPMA feed ratio of 45%, the poly(PMA-*co*-ODMAPMA) hydrogel reached an adhesive strength of up to 78.7 ± 3.8 kPa. However, both higher and lower monomer ratios beyond the optimal range resulted in significantly compromised adhesion performance. This observation is well justified by the fundamental requirement for a balanced thermal-softening capability and inherent mechanical strength when developing high-performance adhesive hydrogels. Specifically, an excessively high DMAPMA ratio compromises the materials’ mechanical strength, while an insufficient ratio hinders effective thermal softening at elevated temperatures.

To further examine the adhesive properties of the poly(PMA-*co*-ODMAPMA) hydrogel, the contact temperature was also investigated. As shown in Figure 5c, the same hydrogel exhibited pronounced temperature-dependent adhesion properties. Following a 10 min heating period at the prescribed temperature, the adhesive strength of the hydrogel increased markedly, rising from nearly 0 kPa at an ambient temperature to approximately 80 kPa at 80 °C. These results demonstrate that the adhesive strength of the hydrogel is attributed to the transition of the hydrogel into a highly elastic state, where increased thermal energy induces softening of the polymeric network, resulting in increased gel softening at elevated temperatures, enabling improved conformational adaptation to the surface topography of diverse substrates. This enhanced interfacial compatibility facilitates superior contact area and mechanical interlocking interactions and, consequently, greater adhesive performance.

### 2.4. Anti-Bacterial Performance

**Figure 6:**
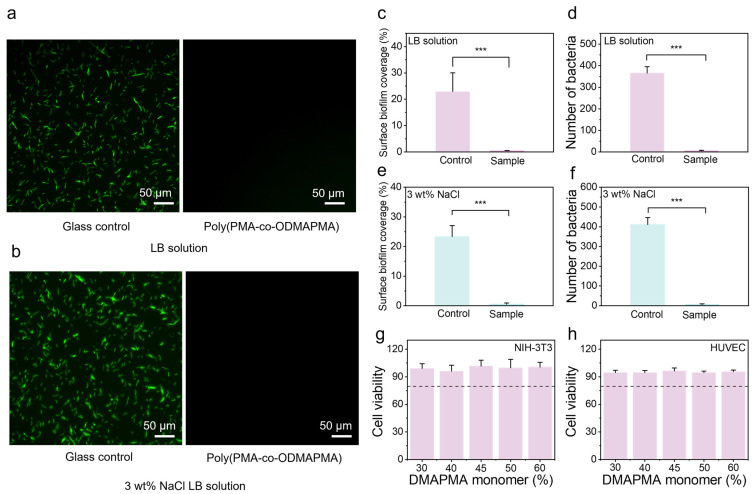
Fluorescent microscope images of biofilm coverage with poly(PMA-*co*-ODMAPMA45%) hydrogel and glass as control in (**a**) LB solution and (**b**) 3 wt% NaCl LB solution. Scale bar: 50 μm. Quantitative analysis of biofilm coverage levels with poly(PMA-*co*-ODMAPMA45%) hydrogel and glass as control in (**c**) LB solution and (**e**) 3 wt% NaCl LB solution. Colony counts and biofilm coverage levels with poly(PMA-*co*-ODMAPMA45%) hydrogel and glass as control in (**d**) LB solution and (**f**) 3 wt% NaCl LB solution. Cell viability of (**g**) NIH-3T3 cells and (**h**) HUVEC cells co-cultured with different gels. Control group: untreated. Data are expressed as means ± SD, n = 3. Statistical significance among various groups was determined by one-way ANOVA with LSD test (*** *p* < 0.001). Quantitative analysis of biofilm coverage levels used poly(PMA-*co*-ODMAPMA45%) hydrogel and glass as control.

Zwitterionic hydrogels have demonstrated exceptional properties and significant application potential in marine anti-fouling. Their high-density ionic groups facilitate the formation of a nanoscale dense hydration layer via strong hydrogen bonding, which effectively impedes the nonspecific adsorption of bacteria, algae, barnacles, and other marine organisms [43,44,45]. This mechanism suppresses biofilm formation and larval settlement, achieving a high anti-fouling efficiency. Here, the biofouling resistance of the samples was evaluated through bacterial adhesion tests. As shown in Figure 6a, strong green fluorescence, indicating dense surface biofilm formation, was observed on the glass surfaces in the control group, revealing a substantial accumulation of bacterial colonies. In striking contrast, the poly(PMA-*co*-ODMAPMA) zwitterionic hydrogel exhibited a remarkable reduction in bacterial adhesion, with only minimal fluorescence detected. Meanwhile, the poly(PMA-*co*-ODMAPMA) hydrogel demonstrated a similar trend in the presence of high salt concentrations. This dramatic decrease in biofilm formation clearly demonstrates the exceptional antibacterial and anti-fouling properties of the poly(PMA-*co*-ODMAPMA) hydrogel, attributed to its zwitterionic nature, which effectively repels microbial attachment and prevents biofilm development. NIH 3T3 and HUVEC are commonly used cell types for biosafety evaluation [46,47,48]. To further ensure the potential of the gel for biomedical translation, the Alamar Blue test was employed in this study to guarantee its safety in biological applications. As shown in Figure 6g,h, the gel exhibited no cytotoxicity to either cell type.

## 3. Conclusions

In this study, a novel tough and thermo-responsive zwitterionic hydrogel was developed by freezing the three-dimensional network under service conditions. On the one hand, the hydrogel demonstrates high strength, ensuring its further application in practical applications. On the other hand, the hydrogel displays significant temperature-dependent mechanical properties, exhibiting enhanced softness and stretchability when heated due to the thawing of the hydrogel’s network. This feature facilitates its adhesion to substrates, further endowing it with high application potential. Moreover, the hydrogel also exhibits satisfactory anti-fouling performance. In summary, this study not only establishes a fundamental platform for the practical employment of zwitterionic hydrogels, but also provides insightful design principles for the innovation of other hydrogel systems for broader application scenarios.

## 4. Materials and Methods

### 4.1. Materials

Phenyl methacrylate, ethylene glycol dimethacrylate, and α-ketoglutaric acid were purchased from Aladdin (Shanghai, China). *N*-(3-dimethylaminopropyl) methacrylamide and 2,4,6-trimethylbenzoyldiphenyl phosphine oxide (TPO) were purchased from Aladdin (Shanghai, China). Hydrogen peroxide was purchased from Xilong Scientific (Shantou, China), and BL21 (DE3) was purchased from Thermo Scientific (Waltham, MA, USA). All organic solvents were analytical grade, and water was purified with a Millipore system (Merck Millipore, Billerica, MA, USA) combining an inverse osmosis membrane and ion exchange resins for synthesis, purification, and swelling tests.

### 4.2. Preparation of Poly(PMA-co-ODMAPMA) Hydrogel

Prescribed amounts of α-ketoglutaric acid, initiator of DMAPMA, PMA, and EGDMA at 0.2 mol% (molar ratio of total monomer) and 0.3 mol% (molar ratio of total monomer) were mixed into a centrifuge tube, and the resulting gel precursor solution was homogenized in an ultrasonic cleaner and poured into a pair of glass pieces separated by a 1 mm silica gel septum. To ensure the high efficiency of the radical polymerization reaction and the uniformity of the hydrogel structure, we performed the reaction under ultraviolet light (wavelength = 365 nm) in a glove box for 8 h. The polymerized gel was placed in a large amount of deionized water to displace the remaining monomers, and the swelling was set at a time point and weighed until the mass leveled off to obtain an equilibrium gel. The oxidation process was the same as that used in a previously reported work, with a slight modification [49]. Generally, the obtained hydrogel was oxidized in hydrogen peroxide solution (30%) to oxidize the dimethylamino group in DMAPMA and change its charge density, and it was finally swelled in pure water. The details of the hydrogel synthesis are listed as follows (Table 1).

### 4.3. Mechanical Properties of Poly(PMA-co-ODMAPMA) Hydrogel

All mechanical properties of the hydrogel under different conditions were recorded by a commercial tensile testing machine equipped with a 100 N load cell (AGX-V 100 N model, Shimadzu Co., Tokyo, Japan). Dumbbell-shaped hydrogels were cut from smooth hydrogel sheets with initial dimensions of 12 mm in length and 2 mm in width. Before each test, the thickness of the sample was measured with a vernier caliper. The strain rate for tensile strength studies at room temperature was 100 mm/min, and at least three parallel samples were recorded for each hydrogel. A temperature-varying tensile test taking place in a water bath was performed on each specimen. The Young’s modulus E, the fracture stress σ_b_, and the tensile work W were calculated from the uniaxial tensile stress–strain curve. E was calculated from the initial slope of the stress–strain curve when the strain was less than 10%, and W was calculated from the integral of the area under the load–extension curve.

### 4.4. Adhesion Properties of Poly(PMA-co-ODMAPMA) Hydrogel

The hydrogels were heated when implementing adhesion (uniform diameter of 10 mm, cross-sectional area of 78.5 square mm) and the substrate in the swelling solvent was set to a certain temperature to avoid this problem. Before testing, the hydrogels and the glass plate were heated to a specific temperature in the presence of solvent for 10 min to allow good contact and infiltration between the gel and the substrate, and then dried water was removed and cooling to room temperature took place. The AGX-V Shimadzu stretcher was then used at a speed of 50 mm/min to stretch until the substrate was detached from the gel. Before the test, the remaining moisture on the surface of the sample was wiped with dust-free paper, and each sample was wiped at least three times. The uniaxial tensile test was performed to measure the adhesion force of the gel to the substrate. The adhesion force was determined by the ratio of the maximum load during stretching to the cross-sectional area of the gel.

### 4.5. Content of Poly(PMA-co-ODMAPMA) Hydrogel

The water content of the hydrogel can be quantified by a swelling assay. The weights of the swelled and dried hydrogels were *W_t_* and *W_0_*, respectively. The water content of the hydrogel was calculated according to the following formula:

(1) $$Water\;content(\%)=\frac{W_{t}-W_{0}}{W_{t}}\times 100\%.$$

### 4.6. Rheological Testing of Poly(PMA-co-ODMAPMA) Hydrogel

The rheometer model used was TA ARES G2 (TA Instruments, New Castle, DE, USA). The rheological behavior of the swelling equilibrium hydrogel was analyzed by the oscillatory temperature mode. The test was performed in the linear region, the frequency was fixed at 1 Hz, the strain was set at 0.1%, the heating rate was 5 °C/min, and the temperature range was 10 °C to 100 °C. The tested samples were hydrogel specimens that were cut into circles and tested in the presence of solvent. The temperature corresponding to the Tanδ peak can be interpreted as the softening temperature of different hydrogels.

### 4.7. FT-IR Spectra of Poly(PMA-co-ODMAPMA) Hydrogel

FT-IR spectra were obtained using a Nicoletis 50-Raptir spectrometer (Thermo Scientific, Waltham, USA). The resultant gel was cut into discs, the position was aligned with the light spot, and the stuck gel was scanned with ATR mode. The wavenumber of the spectrum was set at between 4000 cm^−1^ and 500 cm^−1^, and the resolution was 1 cm^−1^. Each sample was scanned three times.

### 4.8. Biofouling Test of Poly(PMA-co-ODMAPMA) Hydrogel

BL21 (DE3) was selected as the representative bacteria to verify the anti-fouling ability of the poly(PMA-*co*-ODMAPMA) hydrogel. BL21 (DE3) was incubated with LB solution and 3 wt% NaCl LB solution at 37 °C, with shaking at 200 rpm in a biological incubator for resuscitation. Additionally, a bare glass slide (as a control) and poly(PMA-*co*-ODMAPMA) hydrogel were sterilized under UV light for 30 min. Each sample was immersed in the prepared BL21 (DE3) bacterial strain. After 2 days at 30 °C, bacterial BL21 (DE3) attachment and biofilm growth were characterized using fluorescence microscopy (N-STORM, Nikon, Tokyo, Japan). Biological fouling was reported as the cell biofilm coverage calculated by ImageJ software (https://imagej.net/ij/), and the calculation was repeated three times for each condition.

### 4.9. Biocompatibility of Poly(PMA-co-ODMAPMA) Hydrogel

The viability of NIH-3T3 cells and HUVEC cells was measured by the Alamar blue kit after incubation with hydrogels. The NIH 3T3 and HUVEC cells were cultured in DMEM supplemented with 10% (*v*/*v*) FBS and 1% (*v*/*v*) penicillin/streptomycin and incubated in an incubator at 37 °C with a 5% CO_2_ environment. The sterilized gels were placed into a 24-well plate. Then, the cells were paved into a 24-well plate with a density of 2 × 10^4^. The plates were placed inside a cell incubator. After 24 h of co-incubation, the Alamar blue assay was used, following the manufacturer’s instructions. The absorbance values at 570 nm and 600 nm were measured by a UV–vis spectrophotometer. The cell viability rate (*Vc*) was calculated by the following equations:

(2) $$Vc=\frac{\left(117216\times A_{s(570\mathrm{nm})})-(80586\times A_{s(600\mathrm{nm})}\right)}{\left(117216\times A_{c(570\mathrm{nm})})-(80586\times A_{c(600\mathrm{nm})}\right)}\times 100\%$$

where A_s_ and A_c_ are the absorbance levels of the sample and control groups, respectively.

### 4.10. Thermal Analysis

The Differential Scanning Calorimetry (DSC) test was conducted on poly(PMA-*co*-ODMAPMA) samples by raising the temperature from 5 to 100 °C at a heating rate of 10 °C/min using DSC2500 (TA Instruments, New Castle, DE, USA). During the test procedure, the DSC sample was cooled down to 5 °C and heated up to 50 °C, the temperature was then decreased to 5 °C, followed by a second heating phase up to 100 °C.

### 4.11. Elemental Analysis

The solid sample of poly(PMA-*co*-ODMAPMA) was ground into a homogeneous powder and analyzed using an Elementar UNICUBE EA instrument(Elementar, Langenselbold, Germany) in CHNS mode with the following parameters: a combustion temperature of 1150 °C, a reduction temperature of 850 °C, an oxygen flow rate of 20–50 mL/min, and a helium flow rate of 100–200 mL/min. For O mode, the pyrolysis temperature was set to 1150 °C with a helium flow rate of 100–200 mL/min. The sample was introduced into a high-temperature combustion tube, and the resulting gaseous products were separated via an adsorption column before being detected by a thermal conductivity detector to determine the elemental composition percentages.

### 4.12. Statistical Analysis

Statistical analysis of data from the experiments was carried out using SPSS statistical software 27.0 (IBM, Armonk, NY, USA) and one-way ANOVA. All data are presented as the mean ± standard deviation (mean ± SD). Experimental data were regarded as significantly different and were statistically significant when *p* < 0.001 (***) in the results. All data can be found in the main text.

## Figures and Tables

**Table 1:** Experiment details of hydrogel synthesis ^a^.

Entry	Nomenclature	Hydrophobic Monomer		Hydrophilic Monomer		Initiator	Crosslinker
		n (mmol)	m (g)	n (mmol)	m (g)	n (mmol)	n (mmol)
1	poly(PMA-*co*-ODMAPMA 30%)	35	5.68	15	2.55	0.15	0.1
2	poly(PMA-*co*-ODMAPMA 42.5%)	28.75	4.66	21.25	3.62	0.15	0.1
3	poly(PMA-*co*-ODMAPMA 45%)	27.5	4.46	22.5	3.83	0.15	0.1
4	poly(PMA-*co*-ODMAPMA 47.5%)	27.25	4.26	23.75	4.04	0.15	0.1
5	poly(PMA-*co*-ODMAPMA 50%)	25	4.05	25	4.25	0.15	0.1
6	poly(PMA-*co*-ODMAPMA 55%)	22.5	3.65	27.5	4.68	0.15	0.1
7	poly(PMA-*co*-ODMAPMA 60%)	20	3.23	30	5.11	0.15	0.1

^a^: All the samples were prepared with the EDGMA as a chemical crosslinker and α-keto as a photo-initiator.

## Data Availability

The data that support the findings of this study are available from the corresponding author upon request.

## References

[1] Xu X., Guo S., Vancso G.J. (2025). Perceiving and Countering Marine Biofouling: Structure, Forces, and Processes at Surfaces in Sea Water Across the Length Scales. Langmuir.

[2] Banerjee I., Pangule R.C., Kane R.S. (2011). Antifouling Coatings: Recent Developments in the Design of Surfaces That Prevent Fouling by Proteins, Bacteria, and Marine Organisms. Adv. Mater..

[3] Caruso G. (2020). Microbial Colonization in Marine Environments: Overview of Current Knowledge and Emerging Research Topics. J. Mar. Sci. Eng..

[4] Wang X., Jiang Q., Han D., Chen Y., Liu W., Pei Y., Duan J., Hou B. (2025). Progress in anti-biofouling materials and coatings for the marine environment. J. Environ. Sci..

[5] Li Y., Ning C. (2019). Latest research progress of marine microbiological corrosion and bio-fouling, and new approaches of marine anti-corrosion and anti-fouling. Bioact. Mater..

[6] Li S., Feng K., Li J., Li Y., Li Z., Yu L., Xu X. (2024). Marine antifouling strategies: Emerging opportunities for seawater resource utilization. Chem. Eng. J..

[7] Zeng L., Lin X., Li P., Liu F.-Q., Guo H., Li W.-H. (2021). Recent advances of organogels: From fabrications and functions to applications. Prog. Org. Coat..

[8] Qian P.-Y., Cheng A., Wang R., Zhang R. (2022). Marine biofilms: Diversity, interactions and biofouling. Nat. Rev. Microbiol..

[9] Ali A., Culliton D., Fahad S., Ali Z., Kang E.-T., Xu L. (2024). Nature-inspired anti-fouling strategies for combating marine biofouling. Prog. Org. Coat..

[10] Wang Z., Zhang H., Chu A.J., Jackson J., Lin K., Lim C.J., Lange D., Chiao M. (2018). Mechanically enhanced nested-network hydrogels as a coating material for biomedical devices. Acta Biomater..

[11] Zeng L., Liu Z., Huang J., Wang X., Guo H., Li W.-H. (2022). Anti-Fouling Performance of Hydrophobic Hydrogels with Unique Surface Hydrophobicity and Nanoarchitectonics. Gels.

[12] Zeng L., Cui H., Peng H., Sun X., Liu Y., Huang J., Lin X., Guo H., Li W.-H. (2022). Oleophobic interaction mediated slippery organogels with ameliorated mechanical performance and satisfactory fouling-resistance. J. Mater. Sci. Technol..

[13] Zeng L., Fu Y., Cui H., Zhao Y., Liu Y., Lin X., Chao T., Guo H. (2024). Strong, Smart, and Slippery Organo-gels by Network Glassification. Adv. Funct. Mater..

[14] Zhao C., Zhou L., Chiao M., Yang W. (2020). Antibacterial hydrogel coating: Strategies in surface chemistry. Adv. Colloid. Interfac..

[15] Mehta P., Sharma M., Devi M. (2023). Hydrogels: An overview of its classifications, properties, and applications. J. Mch Behav. Biomed..

[16] Kuzina M.A., Kartsev D.D., Stratonovich A.V., Levkin P.A. (2023). Organogels versus Hydrogels: Advantages, Challenges, and Applications. Adv. Funct. Mater..

[17] Shi Q., Mao J., Cai Y., Gao H., Li S., Cheng D. (2022). Bioinspired ionic hydrogel materials with excellent antifouling properties and high conductivity in dry and cold environments. Polym. Chem..

[18] He G., Liu W., Liu Y., Wei S., Yue Y., Dong L., Yu L. (2025). Antifouling hydrogel with different mechanisms:Antifouling mechanisms, materials, preparations and applications. Adv. Colloid. Interfac..

[19] Li L., Scheiger J.M., Levkin P.A. (2019). Design and Applications of Photoresponsive Hydrogels. Adv. Mater..

[20] Gokhale D., Hamelberg A.F., Doyle P.S. (2024). Multifunctional zwitterionic hydrogels for the rapid elimination of organic and inorganic micropollutants from water. Nat. Water.

[21] Li P., Zeng L., Guo H.-L., Guo H., Li W.-H. (2020). Research Progress in Zwitterionic Hydrogels. Acta Polym. Sin..

[22] Zhang H., Wang F., Guo Z. (2024). The antifouling mechanism and application of bio-inspired superwetting surfaces with effective antifouling performance. Adv. Colloid. Interfac..

[23] He H., Tang Y., Zheng M., Chang Y., Chen H., Wei J., Wu J., Zheng J. (2025). Zwitterionic hydrogels from material design to wound dressing applications. Supramol. Mater..

[24] Qu K., Yuan Z., Wang Y., Song Z., Gong X., Zhao Y., Mu Q., Zhan Q., Xu W., Wang L. (2022). Structures, properties, and applications of zwitterionic polymers. ChemPhysMater.

[25] Gao L., Varley A., Gao H., Li B., Li X. (2025). Zwitterionic Hydrogels: From Synthetic Design to Biomedical Applications. Langmuir.

[26] Zhang J., Chen L., Chen L., Qian S., Mou X., Feng J. (2021). Highly antifouling, biocompatible and tough double network hydrogel based on carboxybetaine-type zwitterionic polymer and alginate. Carbohyd. Polym..

[27] Liu S., Tang J., Ji F., Lin W., Chen S. (2022). Recent Advances in Zwitterionic Hydrogels: Preparation, Property, and Biomedical Application. Gels.

[28] Zheng S.Y., Mao S., Yuan J., Wang S., He X., Zhang X., Du C., Zhang D., Wu Z.L., Yang J. (2021). Molecularly Engineered Zwitterionic Hydrogels with High Toughness and Self-Healing Capacity for Soft Electronics Applications. Chem. Mater..

[29] Liu J., Chen J., Liu S., Li T., Chen Y., Chen L., Cai R., Liao X., Zhao T., Chen Y. (2025). Mechanical Training Drives Structural Remodeling of Zwitterionic Hydrogels. Mater. Horiz..

[30] Fath Dehghan H.N., Abdolmaleki A., Pourahmadi M., Hozori S., Gaeini E., Mousavi S.Y., Arvaneh A.-R., Sadat-Shojai M. (2024). Ultra-strength and anti-freezing zwitterionic hydrogels with high ion conductivity: Effect of the hydrophobic monomer in hydrogels mechanical properties. Polym. Test..

[31] Xiao L., Zheng X., Bai J., Tan J., Meng D., Zhang Z., Liu H., Gong L., Luo S., Ye S. (2025). Ordered Interfacial Water Generated at Poly (ionic liquid) Membrane Surface Imparts Ultrafast Water Transport and Superoleophobicity. J. Am. Chem. Soc..

[32] Bagde S., Rohera B.D. (2024). Modification of the swelling behavior of a hydrophilic polymer as an approach to maintaining a constant gel layer thickness. J. Drug Deliv. Sci. Tec..

[33] Richbourg N.R., Peppas N.A. (2020). The swollen polymer network hypothesis: Quantitative models of hydrogel swelling, stiffness, and solute transport. Prog. Polym. Sci..

[34] Hu J., Zhang D., Li W., Li Y., Shan G., Zuo M., Song Y., Wu Z., Ma L., Zheng Q. (2024). Construction of a Soft Antifouling PAA/PSBMA Hydrogel Coating with High Toughness and Low Swelling through the Dynamic Coordination Bonding Provided by Al(OH)_3_ Nanoparticles. ACS Appl. Mater. Interfaces.

[35] Shen J., Du M., Wu Z., Song Y., Zheng Q. (2019). Strategy to construct polyzwitterionic hydrogel coating with antifouling, drag-reducing and weak swelling performance. RSC Adv..

[36] Hu J.Y., Jiao D., Hao X.P., Kong X., Zhang X.N., Du M., Zheng Q., Wu Z.L. (2023). A Facile Strategy to Fabricate Tough and Adhesive Elastomers by In Situ Formation of Coordination Complexes as Physical Crosslinks. Adv. Funct. Mater..

[37] Lin X., Wang X., Cui H., Ouyang G., Guo H. (2023). A universal strategy for preparing tough and smart glassy hydrogels. Chem. Eng. J..

[38] Lin X., Wang X., Cui H., Rao P., Meng Y., Ouyang G., Guo H. (2023). Hydrogels with ultra-highly additive adjustable toughness under quasi-isochoric conditions. Mater. Horiz..

[39] Wang W., Liu Y., Liu Y., Yang X., Wang X. (2023). Highly sensitive smart hydrogels with pH-tunable toughness via signaling cascade amplification. Giant.

[40] Li F., Wu K., Zhang X., Fu Y., Sun T., Guo H., Wang X., Guo H., Meng Y. (2025). “Frozen” Ionogels with High and Tunable Toughness for Soft Electronics. Small.

[41] Wang Z., Liu X., Zhu M., Yang J., Tao S., Gong Z., Xu M., Pan L. (2025). Recent advances in zwitterionic hydrogels: Structure, applications and challenges. J. Mater. Chem. A.

[42] Yin H., You M., Shi X., Yu H., Chen Q. (2024). New insights into pure zwitterionic hydrogels with high strength and high toughness. Mater. Horiz..

[43] Zhang J., Wu M., Peng P., Liu J., Lu J., Qian S., Feng J. (2022). “Self-Defensive” Antifouling Zwitterionic Hydrogel Coatings on Polymeric Substrates. ACS Appl. Mater. Interfaces.

[44] Wang H., Meng L., Ye Y., Wu J., Zhu S., Liu Y., Li K., Yang X., Wei M., Wang M. (2024). Antibacterial zwitterionic hydrogel for flexible and wearable ultrafast-response strain sensors with low hysteresis. Giant.

[45] Wang Y., He C., Chen C., Dong W., Yang X., Wu Y., Kong Q., Yan B. (2022). Thermoresponsive Self-Healing Zwitterionic Hydrogel as an In Situ Gelling Wound Dressing for Rapid Wound Healing. ACS Appl. Mater. Interfaces.

[46] Tang Z., Meng S., Yang X., Xiao Y., Wang W., Liu Y., Wu K., Zhang X., Guo H., Zhu Y.Z. (2024). Neutrophil-Mimetic, ROS Responsive, and Oxygen Generating Nanovesicles for Targeted Interventions of Refractory Rheumatoid Arthritis. Small.

[47] Liu Y., Yang X., Wu K., Feng J., Zhang X., Li A., Cheng C., Zhu Y.Z., Guo H., Wang X. (2025). Skin-Inspired and Self-Regulated Hydrophobic Hydrogel for Diabetic Wound Therapy. Adv. Mater..

[48] Fu Y., Wang Z., Wu K., Li F., Zhang X., Cui H., Wang X., Guo H., Meng Y. (2025). Bio-inspired multifunctional hydrogels with adhesive, anti-bacterial, anti-icing and sensing properties. Chin. Chem. Lett..

[49] Li B., Jain P., Ma J., Smith J.K., Yuan Z., Hung H.-C., He Y., Lin X., Wu K., Pfaendtner J. (2019). Trimethylamine N-oxide–derived zwitterionic polymers: A new class of ultralow fouling bioinspired materials. Sci. Adv..

